# Feedback differences between upper gastrointestinal and colorectal specialists observing laparoscopic trainee surgeon suturing videos

**DOI:** 10.1016/j.sopen.2025.02.001

**Published:** 2025-02-05

**Authors:** Daigo Kuboki, Teruhiko Unoki, Yuji Kaneda, Yoshitaka Maeda, Kosuke Oiwa, Hironori Yamaguchi, Naohiro Sata, Hiroshi Kawahira

**Affiliations:** a Department of Surgery, Division of Gastroenterological, General and Transplant Surgery, Jichi Medical University, 3311-1, Yakushiji, Shimotsuke-shi, Tochigi, Japan; b College of Foreign Studies, Kansai Gaidai University, 16-1, Nakamiyahigashino-cho, Hirakata-shi, Osaka, Japan; c Medical Simulation Center, Jichi Medical University, 3311-1, Yakushiji, Shimotsuke-shi, Tochigi, Japan; d Department of Information and Management Systems Engineering, Nagaoka University of Technology, 1603-1, Kamitomioka-cho, Nagaoka-shi, Niigata, Japan

**Keywords:** Laparoscopic surgery, Suture techniques, Training, Surgery specialty, Formative feedback

## Abstract

**Background:**

Performing laparoscopic suturing requires quality education. Differences in instruction according to trainer surgeon specialty could affect trainee skill acquisition. This study compares the focus of feedback between Upper gastrointestinal (UGI) specialists and Colorectal (CR) specialists.

**Methods:**

A 13-year postgraduate trainee received online feedback for two laparoscopic suturing procedures videos of “low” and “high” difficulty from 16 surgeons (UGI = 8, CR = 8) who are specialists in laparoscopic surgery and qualified by the Endoscopic Surgical Skill Qualification System of the Japan Society for Endoscopic Surgery. The number of feedback comments was compared between specialist groups for grasping the needle, needle driving, knot tying preparation, and knot tying. Both groups were also surveyed regarding suturing procedures.

**Results:**

The UGI group had significantly more feedback comment varieties for knot tying preparation during the “high” difficulty video (UGI 4.0 ± 2.1 (mean ± SD), CR 1.9 ± 1.4, p < 0.05). According to questionnaire results, the UGI group performed suturing more routinely than the CR group, was more confident, and less stressed about the procedure.

**Conclusion:**

In feedback for laparoscopic suturing videos, the UGI group focused more on the preparatory stage for knot tying than the CR group. This indicates that comment focus differs according to specialty, suggesting that instruction from trainers of multiple specialties is optimal.

**Key message:**

In this study, it was shown that the focus of feedback on laparoscopic suturing procedures differs according to the surgeon's subspecialty. These insights could have important implications for optimizing laparoscopic training programs.

## Highlights

• Online feedback was obtained using laparoscopic suturing videos in surgery.
• The feedback was provided by the upper gastrointestinal and the colorectal surgeons.
• Feedback comments were categorized and quantified.
• Differences in the focus between surgeons' subspecialties were observed.
• The upper gastrointestinal surgeon focused more on preparation for knot tying.

## Introduction

The minimally invasive benefits of endoscopic surgery are widely recognized, and in recent years the technique has spread rapidly worldwide [1]. However, there are a variety of challenges, including indirect visualization, loss of freedom of movement, fixed-port positions, and limited working space [2]. Because of the high skill level requiring different techniques from open surgery [3,4], outcomes vary among surgeons and institutions [5]. Therefore, quality continuing education is necessary for improved laparoscopic suturing [6].

The Japan Society for Endoscopic Surgery (JSES) initiated the Endoscopic Surgical Skill Qualification System (ESSQS) in 2004 to maintain quality and educate trainers [7,8]. This system has improved and standardized laparoscopic surgery [9]. Surgeons qualified by ESSQS are technically skilled and capable of coaching trainees. Supervision of laparoscopic surgery by a qualified surgeon has been shown to improve proficiency and safety [10]. Many surgeries by residents can now be performed under the guidance of ESSQS-qualified surgeons, which may support newly qualified surgeons [11].

Laparoscopic suturing procedures are crucial skills evaluated in the ESSQS certification exam. Candidates are required to submit an unedited video showing the entire sequence from grasping the needle with a needle holder to completing the suture and cutting the thread. This performance is rigorously assessed to ensure that suturing is performed accurately and efficiently as intended by the surgeon, and that knot-tying is executed smoothly. The evaluation criteria are consistent across gastric and colorectal surgeries [12]. Instruction is provided within each surgical specialty area for basic procedures such as suturing. During surgery, when the trainee is required to perform a suturing procedure, the trainer provides advice to the trainee. However, instructional differences for the same procedure could depend on the trainer's gastrointestinal subspecialty. This could influence feedback amount and type from instructors. Therefore, instruction may not be equivalent between different trainers, and trainee skill acquisition could be affected. To the best of our knowledge, no studies have compared feedback from trainers of different specialties. In general, gastric surgery provides more opportunities for suturing than colorectal surgery, particularly for gastric cancer and morbid obesity. The temporary sutures in delta-shaped anastomosis in laparoscopic distal gastrectomy with Billroth-I reconstruction, and intracorporeal anastomosis in gastric bypass procedures are common scenarios [13,14]. In addition, bariatric surgery requiring numerous suturing procedures is performed mainly by upper gastrointestinal surgeons [[14], [15], [16]]. Accordingly, we hypothesized that Upper gastrointestinal (UGI) specialists and Colorectal (CR) specialists differ in their focus when providing feedback on suturing procedures. This study aims to compare feedback from UGI specialists and CR specialists who have observed the same video of laparoscopic suturing.

## Materials and methods

One trainee in a laparoscopic surgery training program received feedback from two groups of trainers who were UGI and CR specialists. Trainers viewed two videos of the trainee's surgical procedures of “low” and “high” difficulty. Then, feedback comments were analyzed and compared between groups. In addition, a questionnaire was used to survey the background of each group regarding suturing procedures.

### Trainers

Eight UGI and CR specialist ESSQS-qualified surgeons each (N_total_ = 16) were included in the study.

### Feedback sessions

An online feedback system reported previously shown to have high usability for trainers was used [17]. Surgery videos prepared in advance were shown using the screen-sharing function. The trainer and the trainee communicated via Zoom (Zoom Video Communications, CA, USA), and feedback was provided in real-time. Trainers could pause or repeat sections of the video. The feedback sessions were recorded using Zoom's built-in function. Feedback with pre-recorded videos of the procedure and remote feedback with online tools have been reported to be reliable compared to face-to-face instruction with no quantitative difference in surgical skill improvement [18,19], so it was considered appropriate as a tool. Feedback for Session 1 was based on “low difficulty” Video 1, and Session 2 feedback from “high difficulty” Video 2.

### Procedure videos

**Fig. 1:**
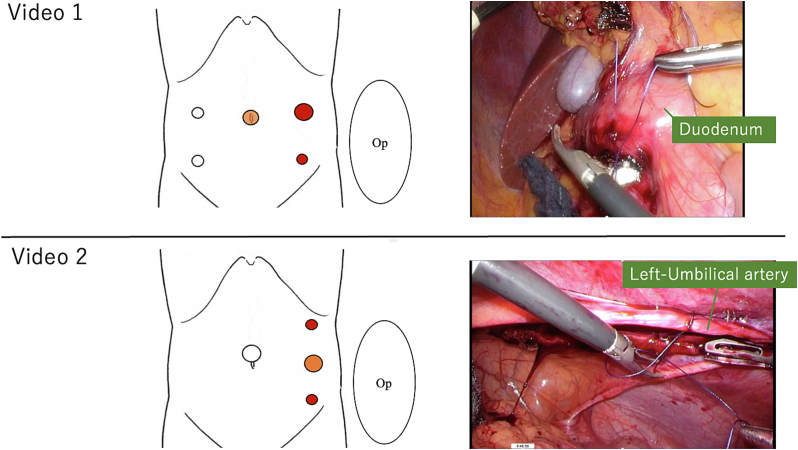
The port sites and the captures of the videos. Op: operator; red circles are the ports for the operator, orange circles are the ports for the camera.

As feedback comments may differ according to procedure difficulty, videos with two difficulty levels were prepared which varied according to the organ sutured, positional relationship with the port, and body position (Fig. 1):

• *Video 1 (3′15″, “low difficulty”):* Repairing the duodenal wall with serosal muscular layer suturing in a scene in which the duodenal serosa was injured during laparoscopic right hemicolectomy. The surgeon performed the procedure standing on the patient's left side with the 5-port according to the general laparoscopic colon resection procedure.
• *Video 2 (3′57″, “high difficulty”):* The stab ligation of the left umbilical artery during laparoscopic ureteral duct resection. The surgeon stood on the patient's left side and performed the procedure through the port on the left anterior axillary line. This was selected because of its difficulty compared to Video 1, as it is a procedure performed facing the ventral side.

In both videos, the procedure was performed by a 13-year postgraduate “intermediate-level” surgeon board-certified by the Japan Surgical Society but still requiring ESSQS qualification from JSES. The video was recorded using an Olympus laparoscopic system (Olympus Medical Systems Corp., Tokyo, Japan). In both scenes, the operator's right-hand forceps were used with a needle holder (KARL STORZ SE & Co. KG, Tuttlingen, Germany), and the left was used with Maryland-type forceps (Olympus Medical Systems Corp., Tokyo, Japan). The threads were made of absorbable violet braided suture, 3–0 VICRYL (ETHICON, Inc., NJ, USA) cut to 13 cm. The scenes involving feedback were not edited, and the same videos were used for all trainers. The videos were not provided to the trainer in advance.

### Feedback classification and counting

The series of suturing procedures were divided into “Phase 1–4” to analyze the statements in detail (Table 1). This classification was initially based on a study by Cuschieri et al. [20] and proposed in a previous study [17]. Statements regarding preparation, instrument selection, anatomy, procedural strategy, and camera work were classified as “Settings,” while comments not classifiable in these categories were “Others.”

**Table 1:** Suture phase classification.

Start		
Phase 1 “Grasping the needle”	Insert the forceps into the abdominal cavity	
	Grasp the needle with the needle holder in the right direction	
Phase 2 “Driving the needle”	Entrance bite	
	Exit bite Remove the needle from the tissue surface	
Phase 3 “Preparation for knot tying”	Pull the suture and make an adequate long tail and short tail	
	Establish the C-loop	
Phase 4 “Knot tying”	Take one or two throws around the forceps	
	Grab the short tail Tie the knot	
Return to Phase 3 until knots are completed.		
End		

The feedback comments in each session were extracted using Adobe Premier Pro (Adobe Inc., CA, USA) subtitling tools. First, imported feedback session videos were edited by Adobe Premiere Pro. Next, trainer comments were extracted and displayed one at a time on the screen as a subtitle. Then, once extracted all subtitles were classified according to the focus of the comment. Finally, comments with the same content were grouped together and the number of varieties tabulated. Video comments were viewed, analyzed, and tabulated by a committee of the co-authors of this study, and disagreements resolved by consensus.

### Questionnaire

After the feedback sessions, trainers accessed the questionnaire via Google Forms (Google LLC, CA, USA). Frequency of suturing procedures per routine surgery was tapped on a four-level scale (0–1 = 1, 2–3 = 2, 4–5 = 3, >5 = 4), and a four-tiered scale with two positive and negative response levels each determined how strongly confident or stressed trainers felt about suturing procedures. Trainers were also asked about the difficulty level of each video on a 5-point scale from 1 = “Very easy” to 5 = “Very difficult,” with 3 as a “neither” neutral response.

### Statistical analysis

The trainers' post-graduation years and feedback time were analyzed using Welch's *t*-test. The number of feedback comment varieties from viewing the video, and responses regarding frequency of suturing procedures by trainers from the questionnaire were analyzed with the Mann-Whitney *U* test. Fisher's exact test analyzed responses regarding trainer gender and confidence level during suturing procedures. Wilcoxon signed-rank test analyzed video difficulty level responses. P-values were statistically significant at <0.05. All statistical analyses were performed with EZR (Saitama Medical Center, Jichi Medical University, Saitama, Japan), a graphical user interface for R (The R Foundation for Statistical Computing, Vienna, Austria) modified from R commander designed for biostatistics [21].

## Results

**Fig. 2:**
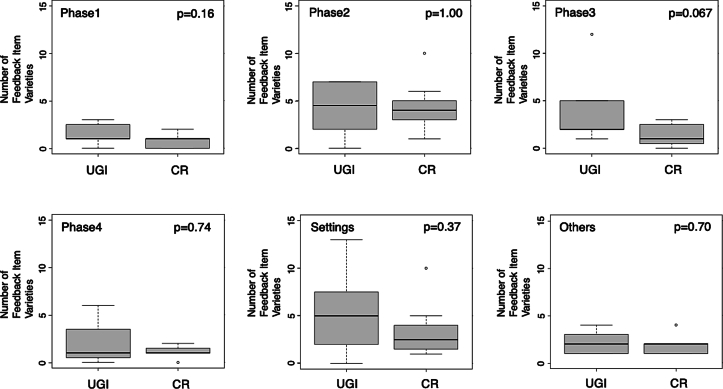
Box plots of the number of feedback comment varieties in Session 1 by the UGI group and CR group. The circles are outliers. The ends of each box represent the 25th and 75th percentiles, and the ends of each line show the maximum and minimum. Lines within boxes represent medians. UGI: upper gastrointestinal group; CR: colorectal group.

**Fig. 3:**
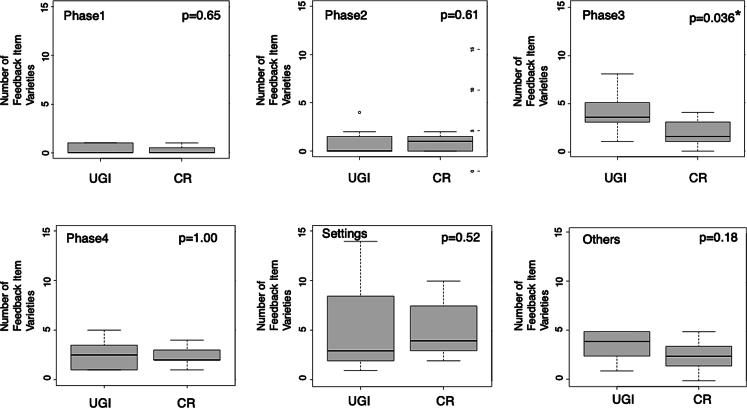
Box plots of the number of feedback comment varieties in Session 2 by the UGI group and CR group. The circle is an outlier. The ends of each box represent the 25th and 75th percentiles, and the ends of each line show the maximum and minimum. Lines within boxes represent medians. UGI: upper gastrointestinal group; CR: colorectal group. *p < 0.05.

Trainer between-group differences for gender (UGI male = 8, CR male = 6 female = 2, p = 0.467) and post-graduate years of practice (UGI 26.63y ±4.66 (mean ± SD), CR 21.75y ±5.61, p = 0.099) were not significant. Between-group feedback time differences for each session were also not significant: Session 1 UGI 1110s ±271.11 vs. CR 949.25 s ±161.74, p = 0.21; Session 2 UGI 985.13 s ±184.48 vs. CR 1097.13 s ±196.92, p = 0.29). The list of extracted feedback comment varieties is shown in Table 2. As shown in Fig. 2, the number of feedback comment varieties in Phase 3 in Session 1 tended to be higher for the UGI group (UGI 3.88 ± 3.60, CR 1.38 ± 1.19, p = 0.067), but the difference was not significant. In Phase 3 of Session 2, however, feedback comment varieties in the UGI group were significantly higher (4.00 ± 2.07) than the CR group (1.88 ± 1.36, p = 0.036, Fig. 3).

**Table 2:** The list of feedback comment varieties for each classification.

	Session 1	Session 2
Phase 1	-Grasping position, direction, and angle of the needle	-Where to grasp the needle in the surgical field
	-How to use left-handed forceps	-Grasping position
		-The number of steps to grasp the needle
Phase 2	-Needle entry point	-Angle of needle insertion
	-Angle of needle insertion	-Direction of driving the needle
	-Depth of driving the needle	-How to remove the needle
	-Direction of driving the needle	-The movement between the first bite and the second bite.
	-Width of driving the needle	-How to use left-handed forceps
	-The movement between the first bite and the second bite.	
	-How to use left-handed forceps	
Phase 3	-Long tail	-Long tail
	-Short tail	-Short tail
	-C-loop	-C-loop
		-Thread deflection
Phase 4	-How to throw around the forceps	-How to throw around the forceps
	-How to grab the short tail	-How to grab the short tail
	-Traction of threads	-Traction of threads
		-Where to perform the procedure in the surgical field
Settings and preparation	-Checking injured area and orientation	-Checking the location to be sutured and orientation
	-Strategies for the procedure	-Strategies for the procedure
	-Equipment to be used	-Equipment to be used
	-Camera	-Camera
	-The movement of the assistant	-The movement of the assistant
		-Physical position of the patient

Questionnaire responses are in Table 3. Suturing procedures in regular surgery were significantly more frequent for the UGI group than the CR group (p < 0.01), and the UGI group was more confident (p < 0.01) and less stressed (p < 0.001) performing these procedures. As for the difficulty of the videos, both the UGI and the CR groups felt that Video 2 was significantly more difficult (p = 0.035 and p = 0.019, respectively).

**Table 3:** Questionnaire results.

Variable	UGI group	CR group	p-Value
“*On average, how many times do you perform suturing in the course of one surgery (0–1 = 1, 2–3 = 2, 4–5 = 3, >5 = 4)?*” mean (± SD)	3.38 ± 1.06	1.25 ± 0.71	0.003^a^


Variables	UGI group		CR group		p-Value
	Strongly yes/yes	No/strongly no	Strongly yes/yes	No/strongly no	
“*Are you good at suturing procedures?*” (n)	8	0	1	7	0.001^b^
“*Do you feel stress during suture ligation?*” (n)	0	8	8	0	<0.001^b^


Variable	UGI group	CR group	p-Value
“*How do you feel about the difficulty of the procedure? (1 = ‘Very easy’ to 5 = ‘Very difficult’)*” mean (± SD)			
Video 1	1.88 ± 0.83	2.75 ± 0.71	0.059^a^
Video 2	3.38 ± 0.92	4.00 ± 0.76	0.222^a^
	p = 0.035^c^	p = 0.019^c^	

Abbreviations: SD, standard deviation.

a Means of scores were compared using the Mann-Whitney *U* test.

b For both groups, “strongly yes/yes” and “no/strongly no” responses were compared using Fisher's exact test.

c Within-group video difficulty levels were compared using the Wilcoxon signed-rank test.

## Discussion

This study investigated differences in feedback focus in laparoscopic suturing procedures depending on gastrointestinal surgeons' specialties. There have been reports on the educational effectiveness of training programs for surgical residents and on the educational effectiveness of suturing procedures using box trainers and virtual reality simulators [22,23]. However, to the best of our knowledge, no studies have compared feedback from trainers across different specialties.

Videos of different difficulty levels – “low” Video 1 for Session 1, and “high” Video 2 for Session 2 – were prepared for observation and feedback from ESSQS-qualified surgeons, and feedback comment varieties were counted for each. Both groups judged Session 2 to be more difficult in the post-feedback questionnaire. During Session 1, the UGI group tended to make numerically more feedback comments about Phase 3 *knot tying preparation* than the CR group, although the difference was not significant. On the other hand, during Session 2 significantly more feedback comments were made about Phase 3, indicating that the UGI group focused feedback on more difficult suturing procedures. For both Session 1 and Session 2, comments were made regarding “long tail,” “short tail,” and “C-loop” formation [20]. There was also an efficiency-conscious remark by the UGI group for the long tail to be grasped properly in one go.

There were two challenging points in Video 2. One is the stab ligation: As the thread is rotated through the tissue after ligation in the first half, adjusting length and angle of the short tail is difficult. Therefore, precise C-loop formation is necessary. The other challenge is that the procedure is performed from an upwards angle toward the ceiling. Patil et al. determined that the best performance for laparoscopic suturing procedures is obtained when the endoscope is viewed from above or on the same plane as the instruments because it is intuitive for the surgeon, while viewing the instruments' plane from below significantly increases the execution time [24]. Furthermore, in such a setting gravity causes threads to twist making them challenging to handle and create C-loops. In the UGI group, there were many feedback comments on eliminating thread twist. From these observations, it can be suggested that the more experienced UGI group made significantly more feedback comments during Session 2 in the preparation stage of knot tying.

In addition, the questionnaire tapped each group's background regarding suturing procedures. The UGI group had significantly more frequent suturing opportunities, indicating they were more experienced with this procedure. The UGI group also showed more confidence and less stress in suturing situations. Other situational data appears to support these results. For example, of 27 instructors at the regular Endoscopic Suture and Ligation Workshop held by JSES, 11 (41 %) are UGI specialists compared to 2 (7 %) CR specialists [25].

Overall, the results of this study showed differences in feedback for laparoscopic suturing procedures according to specialty, meaning there are differences in feedback according to the group to which the trainee belongs. Therefore, it would be beneficial for trainees to receive instruction not only from the same group but also from multiple specialists to ensure uniformity in technique acquisition, including suturing procedure. That is, if the instruction content differs among the supervising surgeons' specialties, there is a possibility of bias in the trainee surgeons' skills.

It is also necessary to address the increasing prevalence of robotic surgery in future. Robotic surgery facilitates suturing procedures due to its features such as 3-D visualization, intuitive motions, and additional freedom of movement [26,27]. Consequently, intracorporeal anastomosis has been increasing in robot-assisted colorectal surgeries [28]. As colorectal surgeons gain more opportunities to perform suturing, confidence in these procedures will likely grow. Consequently, there may be a future generation with no conventional laparoscopic surgery experience. This could result in different feedback patterns compared to the present study. However, the adoption of robotic surgery varies widely across regions, mainly due to economic constraints [29], and it may take time for robotic surgery to completely replace conventional laparoscopic surgery. Therefore, laparoscopic surgical education will remain important for the foreseeable future, and we believe that the findings of our study will have continued relevance in gastrointestinal surgical practice.

Results here are limited to an intermediate-level trainee with previous experience in forceps control, so results may not apply to the entire population of trainees, especially at the novice level with no prior experience.

## Conclusion

While not performed in all laparoscopic surgeries, if necessary, surgeons should be able to do intracorporeal suturing expeditiously. This study may be the first to investigate differences in the instruction of laparoscopic suturing procedures based on subspecialty of gastrointestinal surgeons. The UGI group focused more than the CR group on the preparatory stage of knot tying, indicating that the focus differed depending on the surgeons' specialty. Results from our study suggest that instruction come from multiple specialists to ensure uniformity in technique acquisition.

## CRediT authorship contribution statement

**Daigo Kuboki:** Writing – review & editing, Writing – original draft, Visualization, Validation, Supervision, Software, Resources, Project administration, Methodology, Investigation, Formal analysis, Data curation, Conceptualization. **Teruhiko Unoki:** Writing – review & editing, Writing – original draft, Visualization, Validation, Supervision, Software, Resources, Project administration, Methodology, Investigation, Formal analysis, Data curation, Conceptualization. **Yuji Kaneda:** Writing – review & editing, Writing – original draft, Visualization, Validation, Supervision, Software, Resources, Project administration, Methodology, Investigation, Formal analysis, Data curation, Conceptualization. **Yoshitaka Maeda:** Writing – review & editing, Writing – original draft, Visualization, Validation, Supervision, Software, Resources, Project administration, Methodology, Investigation, Formal analysis, Data curation, Conceptualization. **Kosuke Oiwa:** Writing – review & editing, Writing – original draft, Visualization, Validation, Supervision, Software, Resources, Project administration, Methodology, Investigation, Formal analysis, Data curation, Conceptualization. **Hironori Yamaguchi:** Writing – review & editing, Writing – original draft, Visualization, Validation, Supervision, Project administration, Methodology, Investigation, Formal analysis, Data curation, Conceptualization. **Naohiro Sata:** Writing – review & editing, Writing – original draft, Visualization, Validation, Supervision, Project administration, Methodology, Investigation, Formal analysis, Data curation, Conceptualization. **Hiroshi Kawahira:** Writing – review & editing, Writing – original draft, Visualization, Validation, Supervision, Software, Resources, Project administration, Methodology, Investigation, Formal analysis, Data curation, Conceptualization.

## Ethical approval statement

This study was approved by the Shin-Oyama City Hospital Ethics Review Committee (No. SOK2023-004).

## Funding sources statement

This research did not receive any specific grant from funding agencies in the public, commercial, or not-for-profit sectors.

## Declaration of competing interest

The authors declare that they have no known competing financial interests or personal relationships that could have appeared to influence the work reported in this paper.

## Data Availability

The data that support the findings of this study are available from the corresponding author, Daigo Kuboki, upon reasonable request.
